# Live and let live: Residents' perspectives on alcohol and tobacco (mis)use in residential care facilities

**DOI:** 10.1111/opn.12508

**Published:** 2022-10-13

**Authors:** Lisette de Graaf, Tineke Roelofs, Meriam Janssen, Katrien Luijkx

**Affiliations:** ^1^ Department of Tranzo, School of Social and Behavioral Sciences Tilburg University Tilburg The Netherlands; ^2^ Mijzo Waalwijk The Netherlands

**Keywords:** care of older people, long‐term care, nursing home care, person‐centred care

## Abstract

**Background:**

Alcohol and tobacco use could cause health problems in older adults. Older adults who become in need of 24/7 care due to physical and/or neurological disabilities may need to move to a Residential Care Facility (RCF). RCFs aim to provide person‐centred care (PCC) to enhance quality of life (QoL) of residents.

**Objectives:**

This study aims to explore perspectives of residents on alcohol and tobacco use, which is essential to provide PCC.

**Methods:**

A qualitative research design was chosen, and semi‐structured interviews were conducted. Residents who use alcohol and/or tobacco and those who do not use these substances were purposively selected in two organisations on two types of units: psychogeriatric units and units providing care for residents with mainly physical disabilities. The results were analysed using thematic analysis.

**Results:**

Thematic analysis resulted in five themes: Current use and self‐reflection, knowledge and attitudes, addiction or habit, policies and availability, dependency versus autonomy.

**Conclusion:**

Residents in this study value their autonomy regarding alcohol and tobacco use. They experience dependency on their (in)formal caregivers to use these substances and acknowledge that their use could cause a nuisance to others, challenging the ability of caregivers to implement PCC. Future research could assess how to integrate providing PCC to residents by offering choices and autonomy, while considering the addictive component of these substances, health and safety risks for all.

**Implications for practice:**

This study could help care professionals to become aware of the habits and wishes of residents regarding alcohol and tobacco use and to discuss the possibilities and limitations within RCFs.

**Summary statement of implications for practice**

**What does this research add to existing knowledge in gerontology?**

This research adds knowledge of the perspectives of residents living in residential care facilities (RCFs) regarding alcohol and tobacco (mis)use: their habits, wishes, knowledge, attitudes and experiences regarding this topic.

**What are the implications of this new knowledge for nursing care with older people?**

The findings of this study help to increase the ability of care professionals to provide person‐centred care regarding alcohol and tobacco use in RCF residents.

**How could the findings be used to influence policy or practice or research or education?**

The findings could be used as a foundation for practice and future research to assess how to offer autonomy to residents, while, considering the addictive component, health and safety risks.

## 1 INTRODUCTION

Drinking alcohol and smoking tobacco may cause adverse health outcomes (Burruss et al., 2015; Kulak & LaValley, 2018) Older adults are more sensitive to the negative effects of alcohol, such as cancer, diabetes mellitus and cerebrovascular diseases, due to the biological changes associated with ageing (Galluzzo et al., 2012; Rossow & Træen, 2020). Besides the normal ageing process, these negative effects could be strengthened by an increased use of prescriptive medication (Butt et al., 2020). However, older adults show a low motivation to quit drinking alcohol, according to Burruss et al. (2015), possibly because they do not view alcohol use as potentially problematic and perceive it as a part of long‐standing habits. The adverse health outcomes caused by smoking are also well‐known (Kulak & LaValley, 2018), exemplified as a higher risk of lung cancer, cardiovascular mortality, acute coronary events and stroke (Peto et al., 2000). Moreover, there is a range of short‐term and long‐term advantages of smoking cessation, even in older adults, such as a decreased risk of lung cancer and respiratory infection, a decreased blood pressure and improved lung function. The motivation to quit smoking in older adults is also low (Kulak & LaValley, 2018), likely since nicotine, the addictive component of tobacco causes craving and withdrawal effects after smoking cessation (Tiwari et al., 2020).

Residential care facilities (RCFs) provide 24/7 long‐term care for older adults who may become in need of intensive care due to physical or neurological decline. RCF residents spend the final phase of their life in this facility (Allers & Hoffmann, 2018). With this in mind, RCFs aim to provide person‐centred care (PCC) to enhance quality of life (QoL) and wellbeing of residents (Edvardsson et al., 2008; Li & Porock, 2014). The model of PCC is holistic, enabling residents to contribute to their own care through shared decision‐making and tailoring daily care to their life experiences, abilities and preferences (McCormack & McCance, 2006; Mitchell & Agnelli, 2015; Rosemond, 2009). Next to improving quality of care for residents, providing PCC enhances job satisfaction for care professionals (Rosemond, 2009). In the Netherlands, the average time of living in an RCF is between less than three months and up to two years in RCFs (Zorg Instituut Nederland, 2021) and the population represents a cross‐section of general society as care is almost exclusively publicly funded (Bakx et al., 2020). In line with the PCC principles, the Care and Compulsion Act [Wet Zorg en Dwang], had been in effect in the Netherlands since January 2020 (Ministerie van Volksgezondheid, 2021). Through this act restraints on the freedom of residents living in RCFs have been diminished; involuntary care (e.g., care against the will of a person, including restrictions) has been made illegal unless there is a risk of serious harm to an individual resident, other residents or staff. This includes daily restraints, among others what residents want to eat or drink (Ministerie van Volksgezondheid, 2021; Van der Meulen et al., 2018). Other countries, such as Australia, the United Kingdom and the United States of America, also include legal aspects of residents' rights: the right to a ‘dignified existence’ and ‘self‐determination’ (Grossi et al., 2021). However, the Care and Compulsion Act explicitly protects the autonomy of residents and, in this way, centralises the residents' rights in the daily residential care.

As Dutch RCF residents represent a cross‐section of the general society, part of the residents smoke tobacco and/or drink alcohol and may wish to continue their habits in the RCF. However, these habits could cause conflicts with other residents and staff, as smoking may cause safety risks, such as fire hazards and drinking alcohol could gradually cause a nuisance to other residents and staff. These possible dilemmas and conflicts challenge the ability of care professionals to balance residents' rights and autonomy, which are essential to implement PCC, with the health and safety of all residents (Holmes et al., 2019). It is important to assess the perspectives of residents to enable care professionals to adequately balance these to individual and group interests. To the best of our knowledge, this perspective is lacking in the current literature. The aim of this explorative study is to understand what experiences, knowledge and attitudes are present in residents regarding drinking alcohol and smoking tobacco. This study assesses three research questions: (1) What are the habits and wishes of residents regarding alcohol and tobacco use? (2) How are their habits and wishes regulated or facilitated in RCFs? (3) What are their knowledge and attitudes towards this use?

## 2 MATERIALS AND METHODS

A qualitative research design was chosen to answer the research questions. Semi‐structured interviews were used to gather in‐depth information from residents themselves. Ethical approval was granted by the Ethics Review Board from the Tilburg University School of Social and Behavioural Sciences (Reference: RP39) and approval was obtained from the executive boards of the two participating organisations. The COREQ checklist was used to report the methods and findings of this study (Tong et al., 2007).

### 2.1 Sample

Participants were recruited in two organisations located in the south of the Netherlands, who participate in the Academic Collaborative Centre (ACC) (Luijkx et al., 2020). Both organisations provide mainly complex, continuous 24/7 long‐term residential care for older adults with severe physical and psychogeriatric disabilities and also geriatric rehabilitation, like most RCFs in the Netherlands (Schols et al., 2004). Dutch RCFs are mainly living facilities that do not provide specialised addiction treatment. Participants were recruited on two locations of Stichting Schakelring and one location of Brabantzorg. These organisations have respectively 10 and 33 locations.

Each organisation provided a contact person who enabled the researcher to recruit participants. Participants living in two types of units were included, because these residents are largely dependent on formal caregivers to facilitate and fulfil in their needs, habits and wishes: first, psychogeriatric units, in which care is provided for people with dementia in a moderate to severe stage (Waterschoot et al., 2021). These units are small (8–10 residents) mostly closed wards, designed as group homes with a communal space and individual bedrooms; second, units for people with severe physical disabilities and comorbidities, which are larger (15–20 residents) and are designed as individual apartments, sometimes with a communal space or living area.

Both, alcohol and tobacco use are included in this study. Although, the impact on the environment differs for smoking tobacco and drinking alcohol, both substances cause challenges for care professionals to balance the residents' rights and autonomy with the health and safety of all residents. To explore a broad range of perspectives of residents living in RCFs, residents were purposively selected who use and/or misuse alcohol and tobacco as well as those who do not use alcohol and tobacco or quit in the past. Alcohol misuse is defined as: at least once a week more than six glasses per day for men and more than four glasses per day for women (CBS, 2020). Alcohol use is defined as all other use less then described above. Four units (two per organisation) were included in this study and four participants were selected from each unit. From these four participants, two participants did smoke and/or drink alcohol and two participants did not smoke or drink alcohol. There was no exclusion based on the severity of dementia, physical disability, age or gender. However, residents with severe communication impairments were excluded from this study as participation in an interview would be impossible.

The contact persons of both organisations asked care professionals working on the targeted units to select residents who could be able and willing to participate. Due to their close relationship with the resident, the care professional could estimate whether a resident would like to participate or feel free to refuse participation. The selected residents and the legal representatives of the residents with dementia received an information letter and an informed consent form. Within two weeks of receiving this letter, they were invited to a face‐to‐face or phone meeting to elaborate the information letter. Due to the COVID‐19 pandemic, most meetings prior to the interview were over the phone to limit real‐life contacts. When a resident decided to participate, the interview date was set. Residents with physical impairments were able to provide informed consent themselves. However, residents with dementia are considered legally incapacitated in the Netherlands; therefore, informed consent from a legal representative was necessary and provided (Roelofs et al., 2017).

### 2.2 Data collection

Data collection took place from June 2020 to October 2020. The duration of the interviews varied between 15 and 60 min. One investigator, the first author (LG), conducted the individual interviews at the apartments of the participants or at a conference room at the RCF, depending on the preference of the participant. If the participant or his or her legal representative preferred the presence of the legal representative (usually a family member or close friend), he or she was also present during the interview. This was the case in four of the 16 interviews. The researcher works as a psychologist in one of the participating organisations. Therefore, she is experienced in communication with nursing home residents, including people with dementia and/or people with physical disabilities and comorbidities. However, she was not clinically involved with any of the participants to enable participants to talk freely about their perspectives. The interviews were audio‐recorded and transcribed verbatim. The interviews were semi‐structured using a topic list (Appendix 1).

### 2.3 Data analysis

Thematic analysis was chosen to analyse the data, because it is not bound to a theoretical framework and enables the researcher to unravel and understand the experiences of residents in a broad context (Braun & Clarke, 2006). Qualitative data analysis software (Atlas.ti version 8) was used for the coding process. Three researchers (MJ, TR and LG) coded the first transcript independently and reached consensus on the first emerging codes. Subsequently, one researcher (LG) coded all transcripts and the other two researchers (MJ and TR) each coded half of the transcripts. Discussion among these three researchers on the similarities and differences in coding was maintained throughout the process to reach consensus on the coded version of each transcript. After 12 of 16 transcripts were coded, no new codes were added. This indicated that data saturation was reached. After this first step, data were analysed by grouping codes in Atlas.ti to find patterns which resulted in recurrent themes. Consensus regarding these overarching themes was reached among all researchers and these findings are described in the results.

## 3 RESULTS

Sixteen participants were interviewed, seven women and nine men, aged between 66 and 91 years. In this sample, four participants drink alcohol but do not smoke tobacco; three participants smoke tobacco but do not drink alcohol; two participants use both alcohol and tobacco and seven participants do not smoke nor drink alcohol because they never drank nor smoked or quit before moving to the RCF. The characteristics of the participants are shown in Table 1. Participants with a ‘P’ in their code live in psychogeriatric units and participants with a ‘S’ in their code live in (somatic) units for residents with physical disabilities and comorbidities. All gathered information on smoking tobacco and drinking alcohol is based on the self‐report of participants.

**TABLE 1:** Participant characteristics

Participant code	Gender	Dementia or physical impairments with comorbidities	Smoking tobacco	Drinking alcohol
P1	Female	Dementia	Non‐smoking^a^	Not drinking^b^
P2	Female	Dementia	Non‐smoking	Drinking
P3	Female	Dementia	Non‐smoking^a^	Not drinking^b^
P4	Male	Dementia	Smoking	Drinking
P5	Male	Dementia	Smoking	Not drinking^b^
P6	Female	Dementia	Non‐smoking^a^	Not drinking
P7	Male	Dementia	Non‐smoking^a^	Not drinking^b^
P8	Male	Dementia	Non‐smoking^a^	Drinking
S1	Male	Physical impairments with comorbidities	Non‐smoking^a^	Drinking
S2	Male	Physical impairments with comorbidities	Smoking	Not drinking^b^
S3	Male	Physical impairments with comorbidities	Non‐smoking^a^	Not drinking^b^
S4	Female	Physical impairments with comorbidities	Non‐smoking	Drinking
S5	Male	Physical impairments with comorbidities	Non‐smoking^a^	Not drinking^b^
S6	Female	Physical impairments with comorbidities	Non‐smoking^a^	Not drinking
S7	Male	Physical impairments with comorbidities	Smoking	Not drinking^b^
S8	Female	Physical impairments with comorbidities	Smoking	Drinking

^a^ Participants who used to smoke, but quit.

^b^ Participants who used to drink alcohol, but quit.

The thematic analysis resulted in five overarching themes, that is (1) current use and self‐reflection (2) knowledge and attitudes (3) addiction or habit (4) policies and availability and (5) dependency versus autonomy. The results were similar for participants with dementia and participants with physical impairments and comorbidities except for one topic, that is the experienced dependency on (in)formal caregivers to drink alcohol or smoke tobacco.

### 3.1 Current use and self‐reflection

Nine of the total 16 participants used to smoke but quit, two never smoked, and five participants still smoke (Table 2). Two participants quit smoking, because they were confronted with adverse health outcomes in their environment and based on information provided by health care professionals. One of these participants, who does not smoke nor drink anymore, described: ‘My wife needed heart surgery … They informed us how bad smoking is for your health. I heard it before, but now it became real. So, I quit smoking’. (S3). Eight of the 16 participants used to drink alcohol but quit, two never drank alcohol and six participants still drink alcohol. One participant, who does not smoke nor drink anymore, described why he quit drinking: ‘I swore on the grave of my wife to never drink alcohol again and I didn't … When my wife was dying, she said that I had to quit drinking alcohol, because it would not end well for me’. (P7).

**TABLE 2:** Self‐reported current use

Tobacco		Alcohol	
Number of cigarettes or cigars	*N*	Number of alcoholic consumptions	*N*
>10 cigarettes per day	2	3 units per day	1
10 cigars per day	1	2 units per day	1
4 cigarettes per day	2	1 unit per day	1
		1 unit per week	1
		<1 unit per month	2
Total number of participants who smoke	5	Total number of participants who drink alcohol	6

Participants described why they started smoking or drinking in the past and how they feel about this now looking back. One participant, who does not drink nor smoke anymore, stated: ‘When I was 13, I went to a boarding school. On Sunday we got the opportunity to buy cigarettes for a week, so we did. It looked cool and once I started smoking it became a habit and an addiction’. (S3).

Five of the six participants who drink alcohol described that they are happy with their current use. For example, they described associating it with social events and they like the taste of a glass of wine or beer. A participant described: ‘I know I am not annoying when I drink, so I think it is fine for me to drink alcohol. But I don't want to bother other people’ (P4). When it comes to smoking, four of the five smoking participants wanted to continue smoking. One participant responded: ‘I sit down and a cigar and the tv. I like that … It would be a pity to quit’ (P5). One of the five participants was sceptical and described that it depends on the cigarette whether he wants to quit or continue smoking.

Participants mentioned a decrease in their alcohol or tobacco use when they became older, like S2, who smokes but quit drinking, reported: ‘You smoke less because there are fewer places where you are allowed to smoke … I don't want to smoke more cigarettes than I do now’. Participant P7, who quit smoking and drinking alcohol, reflected on his alcohol use over time: ‘I used to drink a lot when I was in my thirties. I really let my hair down … Later, I only drank occasionally at parties, but it was already less intense’.

### 3.2 Knowledge and attitudes

Some participants mentioned specific experiences in their past that had guided their behaviour regarding smoking and drinking alcohol, as P2, who drinks alcohol but never smoked, described: ‘My father used to smoke, and I hated that smell. So, I thought that I certainly didn't want that’. Moreover, the changed attitude towards smoking over time in general society was experienced by participants; smoking tobacco is not accepted anymore in society: ‘Smoking isn't popular socially seen … You can't compare it with 30 or 40 years ago. When I went to school, everyone smoked. Teachers entered the classroom with a cigar … We had more opportunities to smoke’. (P4, participant who smokes and drinks).

With regard to drinking alcohol, both personal experiences and the social context tended to determine participants' alcohol use: for example, they only drink alcohol in the RCF when they have visitors. In the past, they used to drink alcohol on special occasions, such as parties, in bars or during carnival (a Catholic folk festival held every year, 40 days before Easter). Participant P5, who smokes but quit drinking, stated: ‘In a bar, I don't drink five cups of coffee… Then I like to drink some beers, but not too much’.

Participants shared their knowledge of the consequences of alcohol and tobacco use. The reported health consequences of smoking ranged from coughing a bit to lung cancer. Four of the five smoking participants described negative health consequences of smoking. One participant, who smokes but quit drinking, described: ‘You see the most horrible pictures on cigarette packages … But I don't think people are warned by the pictures and I think that it will not be as bad for your health as shown on the pictures … At least, that's what I think.’ (S2). Three participants, two who smoke and one who never smoked, warned their children about the consequences of smoking.

Participants reported less about the health consequences of drinking alcohol compared to smoking; two of the six participants who drink alcohol described these consequences. Participants focused on other consequences, such as getting drunk, not driving drunk and not drinking when using specific medication or when you have poor health. They also described that you become looser and less alert when drinking alcohol. One participant, who smokes but quit drinking alcohol, advised in general not to start drinking too soon: ‘Don't start drinking too soon … and try not to start drinking at all. When you think you can drink your first beer, you also think you can drink a second and a third … but that's not good … Actually, I don't really miss drinking alcohol.’ (S2). Another participant, who quit smoking and drinking, noted: ‘I think it's strange that alcohol may be as bad as smoking tobacco. However, tobacco is banned, and alcohol is no problem at all.’ (S3).

### 3.3 Addiction or habit

Participants described whether they viewed smoking tobacco and drinking alcohol as an addiction or as a habit. Overall, participants described that drinking alcohol becomes an addiction when you need to have a drink every day and it is a habit when you are used to drinking alcohol daily but do not have the urge to drink alcohol every day. Three participants reported that drinking alcohol is a habit, two of these participants drink alcohol, and one participant quit drinking alcohol. Participant S2, who smokes but quit drinking, described: ‘I think alcohol is a simple kind of drug … tolerated, you can just drink a beer every night, then you might be addicted … Or a habit … I think it is a habit instead of an addiction’. Four participants stated that drinking alcohol could become an addiction. These participants used to drink alcohol but quit. Three of the four participants described addiction as a severe consequence of alcohol use, which is difficult to overcome. For example, S5, who quit drinking and smoking, described: ‘When you drink alcohol … you change … physically and mentally. To overcome an addiction, you must be admitted to a rehabilitation center, but it is still questionable whether you can treat an addiction. I don't think so. I think they should forbid alcohol use, because overall it is horrible’.

Three participants considered smoking tobacco as an addiction, because you cannot quit easily, and the craving to smoke cigarettes persists. From these three participants, two quit smoking and one still smoke. He reported: ‘My neighbor desired to smoke every day and now he started again … He quit ten years ago … Once you start smoking, you cannot quit anymore’ (S2). Five participants described smoking as a habit instead of an addiction. From these five participants, three smoke themselves and two quit smoking. Participant S3, who quit smoking and drinking, reported: ‘Once a cigarette package was opened, you just took a cigarette and smoked … It was a habit’.

### 3.4 Policies and availability

Overall, participants are satisfied with the policies and restrictions to smoke or drink in the RCFs, like S2, who smokes but quit drinking, reported: ‘I understand that it isn't allowed to smoke anywhere you want inside the facility … I'm happy that this facility offers a well‐ventilated smoking area’. Two participants, one who smokes but quit drinking and one who quit smoking and drinking, specifically described that the RCFs should facilitate the opportunity to drink alcohol, even though they both quit drinking alcohol. Participant S5, who quit smoking and drinking, illustrated why he is happy with the policies of the RCF: ‘If people smoke outside, it is okay … I wouldn't appreciate it if you're allowed to smoke inside this facility … Then, I would feel isolated … I would avoid places where others smoke. So, I would be limited in my freedom to move around the RCF’.

On the contrary, participant S8, who smokes and drinks, is not satisfied with the current policies of the RCF where she lives. She has a big apartment but does not have a smoking area. Therefore, she smokes outside or with her husband in his car: ‘There are no indoor smoking areas and that bothers some residents. I've got a beautiful apartment … I don't understand why there is no smoking area’.

### 3.5 Dependency versus autonomy

Despite the overall satisfaction with the policies, participants are dependent on their informal and formal caregivers to both smoke tobacco and drink alcohol. Mainly participants with physical disabilities experienced restrictions on smoking tobacco or drinking alcohol compared to participants with dementia, as S7, who smokes but does not drink alcohol, experienced: ‘It depends how much I smoke … on how I feel and the time there is … The nurses help me to go outside and to light a cigarette … It depends on their time’. Participant P7, who does not drink alcohol nor smoke tobacco anymore, reported: ‘I think you can drink alcohol … If you ask it in the right way … then it's possible, or not. It depends on which nurse you ask’. The participants who currently drink alcohol or smoke tobacco are dependent on their informal caregivers to purchase alcohol or cigarettes.

All participants considered it to be obvious that everyone should decide for themselves to drink alcohol or smoke tobacco, as long as there is no nuisance to others. Participants made a careful consideration: on the one hand, participants were aware of the necessity to protect residents and their social environment against the adverse outcomes of smoking, such as fire. On the other hand, participants described that residents are already limited in their possibilities, and you do not want to take away these final things they enjoy, such as smoking or drinking alcohol. One participant, who smokes but quit drinking, considered that other residents might be bothered: ‘If someone else is bothered, I don't smoke. Look, if someone has asthma, I certainly don't smoke. Even if they are around all day, I will not smoke.’ (S2).

Participants tend to describe different considerations when it comes to alcohol and tobacco use, possibly due to the dichotomous nature of smoking tobacco: one cigarette could cause a nuisance to others, while drinking alcohol leads gradually to a nuisance to the social environment. For example, 6 participants, 2 who smoke themselves and 4 who do not smoke, reported that people should be able to smoke but they are bothered when someone smokes near them, no matter how many cigarettes were smoked. Participants also reported that they accept alcohol use in others. However, 11 participants argue that there is a limitation in the accepted amount of alcohol due to the consequences when people drink too much alcohol and get drunk. Of these 11 participants, four do not drink nor smoke, three do not drink but smoke, two drink but do not smoke and two both drink and smoke.

## 4 DISCUSSION

This empirical study aimed to explore and understand the perspectives of residents living in RCFs concerning their experiences, knowledge and attitudes regarding alcohol and tobacco use. To reach this aim, this study assessed three research questions: (1) What are the habits and wishes of residents regarding alcohol and tobacco use? (2) How are their habits and wishes facilitated or regulated in RCFs? (3) What are their knowledge and attitudes towards this use? The main findings of this explorative study indicate that the participating residents are satisfied with their current use, even though they have experienced a decrease in their alcohol and tobacco use due to their age and their admission to an RCF. They acknowledge possible adverse (health) outcomes due to alcohol and tobacco use. Participants feel it is obvious that everyone should decide for themselves to smoke tobacco or drink alcohol, if there is no nuisance to other people. Overall, residents are satisfied how their use is facilitated by the RCF, but they also recognise dilemmas of smoking and drinking in RCFs. They highly value their autonomy, but they experience dependency on (in)formal caregivers to purchase and use alcohol and tobacco.

The results of this study indicate possible differences between the focus from a public health approach and the participating older adults who spend the final phase of their life in RCFs. Public health services focus increasingly on the prevention of alcohol and tobacco use (Bell et al., 2009), due to the well‐known adverse health outcomes. From this point of view, safety risks for residents themselves and their environment outweigh the autonomy of residents (Grossi et al., 2021). Similar to the public health services, the participating residents tend to acknowledge the health problems of alcohol and tobacco use and, in addition, mentioned a decrease of acceptance in general society regarding smoking tobacco over the past 30 to 40 years. However, the participants prefer to maintain their autonomy regarding their use when living in an RCF and tend to justify their use by arguing that in the time they grew up the use of both substances was part of daily life. This is in line with the studies of Tolvanen and Jylhä (2005) and Wilson et al. (2013), but the research of Cummings and Proctor (2014) showed that the adverse effects of smoking were already well established in the 1960s and, therefore, could be known to the current older population. The claim on autonomy by the participants is in line with Tolvanen and Jylhä (2005), who showed that it is important for older adults to perceive themselves as responsible for their health and as independent to decide for themselves whether alcohol is good or bad for their health. Although it is questionable whether alcohol and tobacco (mis)use is or was a choice, the participants seem to experience that their use increases their QoL and autonomy in their final phase of life.

As this study points out the importance for the participating residents to maintain their autonomy, they also even feel it is obvious that everyone should decide for themselves to drink alcohol or smoke tobacco, which is in line with providing PCC and the Care and Compulsion Act. However, their autonomy is jeopardised due to dependency on their (in)formal caregivers and is complicated by the right for care professionals and other residents to work and live in a safe environment (Grossi et al., 2021). Previous research also reported on the dependency of RCF residents on their (in)formal caregivers to provide daily care and fulfil their needs, habits and wishes (Fazio et al., 2018). The Care and Compulsion Act, in effect since January 2020 in the Netherlands, encourages care professionals to provide PCC by providing choices in the daily life of residents, within the possibilities of the RCFs and avoiding involuntary care and restraints. This act protects the autonomy of RCF residents by law and put them in charge of their own care despite their physical and/or cognitive disabilities.

Although the participating residents prefer to maintain their autonomy, they acknowledge that their wish to use alcohol or tobacco could cause a nuisance to other residents and staff. This potential conflict of interests is inherent to living in a group in an RCF and may cause a dilemma and challenge for caregivers, which was also described by Holmes et al. (2019): on the one hand, caregivers aim to provide residents with choices which may increase their QoL, while on the other hand, caregivers aim to assure the health and safety of all residents.

### 4.1 Limitations

This qualitative study provided an exploration of the perspectives of individual residents living in RCFs regarding alcohol and tobacco use. The sample was small (*N* = 16) and more men than women participated in this study. This sample does not represent residents living in Dutch RCFs: more woman than men live in RCFs in the Netherlands (Verkooijen, 2020). However, data saturation was reached, and the study provides important insight into residents' perspectives regarding alcohol and tobacco use.

Another limitation of this study is that the data are based on self‐report without verification from (in)formal caregivers. In general, underreporting is common in self‐reports of unhealthy behaviour and the true prevalence of alcohol and tobacco use may be higher than reported (Van Der Nagel et al., 2017). However, previous research also found that caregivers may underestimate the prevalence in residents (Dreher‐Weber et al., 2017). The aim of this study was to understand the experience of participants and, therefore, our results are based on self‐reported use without verifying with their caregivers.

## 5 CONCLUSION

Through this study we found an indication that most participating residents who currently smoke or drink do not want to change their behaviour. However, public health services prevail the health and safety of all residents above the residents' autonomy and focus on the prevention of alcohol and tobacco use. Future research could further explore how the two different perspectives could be integrated in RCFs: on the one hand providing PCC to residents at the end of their lives by offering choices and autonomy, while on the contrary considering the addictive aspect of these substances and the health and safety risks of all residents. For example, by exploring the views of (in)formal caregivers on this topic and how they incorporate these perspectives in the daily care of residents.

## 6 IMPLICATIONS FOR PRACTICE

- The findings indicate that it could be helpful for care professionals to assess the habits and wishes of residents regarding alcohol and tobacco use with residents themselves to increase their ability to provide PCC.
- This study points out that it may be helpful for care professionals to discuss the possibilities and limitations regarding alcohol and tobacco use with residents to adapt the context in which care is delivered to each resident.
- When RCFs develop policies regarding alcohol and tobacco use, it could be valuable to incorporate the health, safety and addictive components of alcohol and tobacco and the perspectives of residents.

## CONFLICT OF INTEREST

None.

## Data Availability

The data that support the findings of this study are available on request from the corresponding author. The data are not publicly available due to privacy or ethical restrictions.

## APPENDIX 1

### TOPIC LIST

**Table:**

General questions	How old are you? Where were you born and raised? What are your first thoughts when you hear the word ‘alcohol’? What are your first thoughts when you hear the word ‘tobacco’?
*Smoking tobacco*	
Topic	Example questions
Personal use of tobacco	Do you smoke? Can you tell me more about this? When do you smoke? When do you not smoke? What feeling do you get when you smoke? How many cigarettes do you smoke a day? How have your smoking habits changed over time?
Wishes concerning tobacco	If you could choose, would you like to smoke? Where are you allowed to smoke in this RCF and how is smoking facilitated or regulated? What do you expect regarding the rules about smoking in this RCF?
Reasons to use or not to use tobacco	Could you tell me why you smoke tobacco or why you do not smoke tobacco? Were these reasons similar in the past?
Knowledge of the consequences of tobacco	What do you know about the mental or physical consequences of smoking tobacco? What do you know about the advantages? What do you know about the disadvantages?
Attitude towards tobacco	What do you think when you or people in your environment smoke tobacco?
*Alcohol*	
Topic	Example questions
Personal alcohol use	Do you drink alcohol? Can you tell me more about this? When do you drink alcohol? When do you not drink alcohol? What kind of feeling do you get when you drink alcohol? How have your drinking habits changed over time?
Wishes concerning alcohol use	If you could choose, would you like to drink alcohol? How is drinking alcohol regulated or facilitated in this RCF? What do you expect regarding the rules about drinking alcohol in this RCF?
Reasons to drink or not to drink alcohol	Could you tell me why you drink alcohol or why you do not drink alcohol? Were these reasons similar in the past?
Knowledge of the consequences of alcohol use	What do you know about the mental and physical consequences of drinking alcohol? What do you know about the advantages? What do you know about the disadvantages?
Attitudes towards alcohol use	What do you think when you or people in your environment drink alcohol?
